# Indocyanine green fluorescence to ensure perfusion in middle segment-preserving pancreatectomy: a case report

**DOI:** 10.1186/s40792-021-01344-y

**Published:** 2021-12-20

**Authors:** Tomohiro Iguchi, Norifumi Iseda, Kosuke Hirose, Mizuki Ninomiya, Takuya Honboh, Takashi Maeda, Fumi Sawada, Yu-ichi Tachibana, Tetsuro Akashi, Naotaka Sekiguchi, Noriaki Sadanaga, Hiroshi Matsuura

**Affiliations:** 1 Department of Surgery, Saiseikai Fukuoka General Hospital, 1-3-46 Tenjin, Chuo-ku, Fukuoka, 810-0001 Japan; 2 Department of Surgery and Science, Graduate School of Medical Sciences, Kyushu University, 3-1-1 Maidashi, Higasi-ku, Fukuoka, 812-8582 Japan; 3 Department of Internal Medicine, Saiseikai Fukuoka General Hospital, 1-3-46 Tenjin, Chuo-ku, Fukuoka, 810-0001 Japan

**Keywords:** Middle segment-preserving pancreatectomy, Indocyanine green fluorescence, Blood supply, Pancreatic ductal adenocarcinoma, Intraductal papillary mucinous neoplasm

## Abstract

**Background:**

Middle segment-preserving pancreatectomy (MSPP) is an alternative to total pancreatectomy that allows for the preservation of the endocrine and exocrine functions of the pancreas. However, maintaining perfusion to the pancreatic remnant is of critical importance. We describe the first case to our knowledge in which indocyanine green (ICG) fluorescence was used to confirm perfusion to the pancreatic remnant during MSPP.

**Case presentation:**

A 79-year-old man with diabetes mellitus was referred to our hospital for treatment of a pancreatic tumor. Computed tomography revealed a hypovascular mass in the uncus of the pancreas and dilatation of the main pancreatic duct, measuring 13 mm in the tail of the pancreas. He was diagnosed with cancer of the pancreatic uncus via endoscopic ultrasound and fine-needle aspiration revealed a mixed-type intraductal papillary mucinous neoplasm (IPMN), along with high-risk stigmata in the tail of the pancreas. We performed MSPP and the length of the pancreatic remnant was 4.6 cm. The dorsal pancreatic artery was preserved and perfusion to the pancreatic remnant was confirmed by ICG fluorescence. Histopathological examination showed a pancreatic ductal adenocarcinoma in the uncus (pT1cN1M0, pStage 2B) and IPMN in the tail of the pancreas. The postoperative course was complicated by a grade B pancreatic fistula, but this was successfully treated with conservative management. The patient was transferred to a hospital 33 days after surgery. Insulin administration was necessary, but C-peptide was detectable and blood glucose was relatively well-controlled. He did not exhibit any exocrine dysfunction when pancreatic enzyme supplementation was administered.

**Conclusion:**

ICG fluorescence can be used to evaluate perfusion to the pancreatic remnant during MSPP.

## Background

Pancreatic ductal adenocarcinoma (PDAC) and intraductal papillary mucinous neoplasms (IPMN) with a high risk of malignancy are two potential indications for pancreatectomy [1, 2]. In patients with multiple pancreatic lesions, total pancreatectomy (TP) has occasionally been performed to achieve curative resection [3]. However, metabolic disorders such as insulin-dependent diabetes mellitus (DM), weight loss, and severe diarrhea and malabsorption remain major problems after TP [3]. Middle segment-preserving pancreatectomy (MSPP) was introduced as an alternative to TP that allows for the preservation of pancreatic endocrine and exocrine functions [4], however, perfusion to the pancreatic remnant is critical. Recently, indocyanine green (ICG) fluorescence is increasingly being used for intraoperative blood flow evaluation during gastrointestinal surgery [5]. We report the first case to our knowledge of using ICG fluorescence to confirm perfusion to the pancreatic remnant during MSPP.

## Case presentation

A 79-year-old man was referred to our hospital for further investigation and treatment of a pancreatic tumor detected during impaired glucose tolerance and an evaluation of elevated serum carbohydrate antigen 19-9 (CA19-9). He had no chief complaint, but did have a history of DM that was being treated with dipeptidyl peptidase-4 inhibitor and sulfonylurea. Physical examination revealed no abnormal findings within the abdomen and laboratory examination revealed no anemia or hyperbilirubinemia. Glycated hemoglobin A1C (HbA1C) level was 7.0% and serum levels of carcinoembryonic antigen and CA19-9 were 1.3 ng/ml and 59.3 U/ml, respectively.

**Fig. 1:**
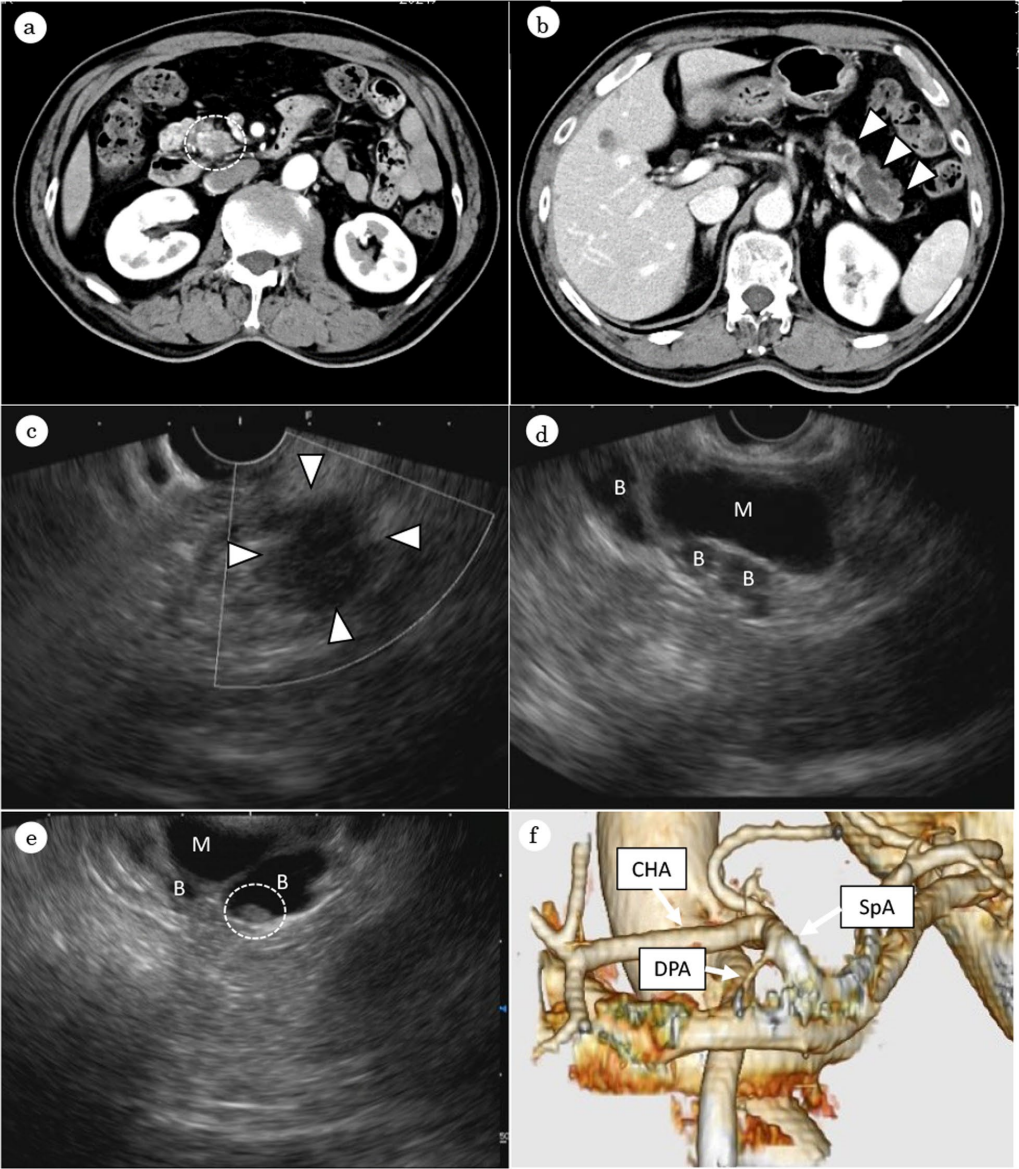
Contrast-enhanced CT revealed a hypovascular mass in the uncus of the pancreas, suspected of pancreatic cancer (encircled by a white dotted line) (**a**). Main pancreatic duct was dilated, 13 mm in size and multiple cystic lesions were also seen, indicating mixed-type IPMN (arrowhead) (**b**). Endoscopic ultrasonography revealed a hypoechoic mass in the uncus of the pancreas (arrowhead) (**c**) and dilatation of the main (M) and branch (B) pancreatic ducts (**d**) with mural nodule, 4.6 mm in size (encircled by a white dotted line) (**e**). Preoperative 3D-CT. The DPA originated from the proximal SpA (**f**). *IPMN* intraductal papillary mucinous neoplasm, *DPA* dorsal pancreatic artery, *SpA* splenic artery, *CHA* common hepatic artery

Contrast-enhanced computed tomography (CT) revealed an ill-demarcated hypovascular mass, 15 mm in diameter, in the uncus of the pancreas (Fig. 1a). There were no findings that suggested vascular invasion or nodal or distant metastasis. Dilatation of the main pancreatic duct and multiple cystic lesions were also seen in the tail of the pancreas, with no mural nodules found (Fig. 1b). Endoscopic ultrasonography (EUS) revealed a hypoechoic mass in the uncus of the pancreas (Fig. 1c). EUS-guided fine-needle aspiration and cytology revealed PDAC. Dilatation of the main and branch pancreatic ducts was present in the tail of the pancreas (Fig. 1d), with a maximum diameter of the main pancreatic duct of 13 mm. These features suggested mixed-type IPMN with high-risk stigmata. A small (4.6 mm) mural nodule in the branch pancreatic duct was also found (Fig. 1e).

**Fig. 2:**
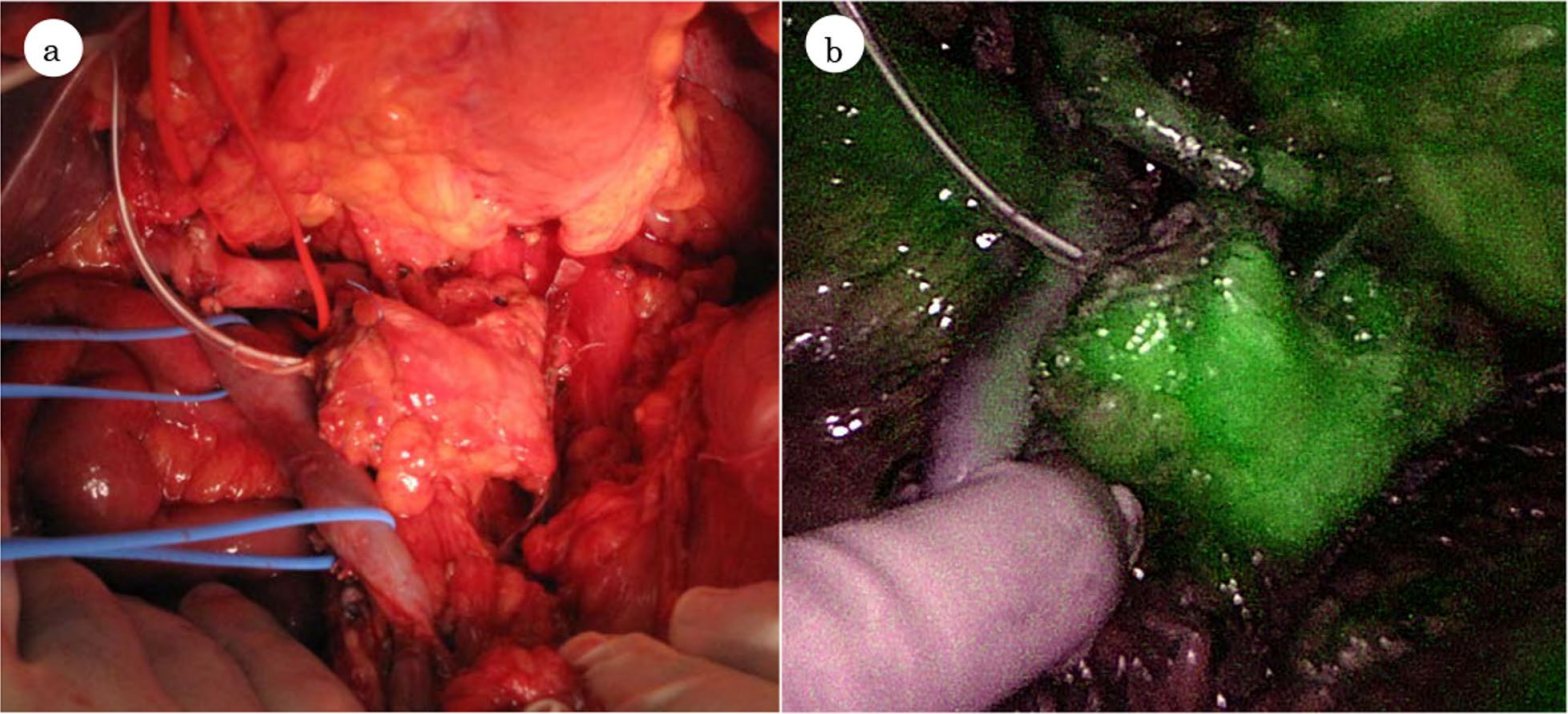
The middle segment of the pancreas, 4.6 cm in size with satisfactory complexion was remaining (**a**). The presence of the pancreatic remnant microperfusion was definitely confirmed by ICG fluorescence (**b**)

We decided to resect IPMN, low-grade malignancy in the tail of the pancreas along with PDAC in the head of the pancreas, because the patient was elderly, but was considered to be fit on geriatric screening and the procedure of additional resection for IPMN would be only total pancreatectomy. Therefore, we elected to perform MSPP alternative to TP owing to the patient’s age, postoperative quality of life, and IPMN tumor grade, while splenectomy was also performed concerning the technical difficulty, time-consuming and perioperative complications related to spleen-preservation. First, we started the pancreaticoduodenectomy procedure and the pancreas was divided at the location of the superior mesenteric vein. The frozen specimen of the pancreatic stump was negative for cancer. We then performed the distal pancreatectomy and splenectomy. The divided line of the distal pancreas was 2 cm on the proximal side of the pancreatic tail tumor. Preoperative CT showed the dorsal pancreatic artery (DPA) branching from the proximal splenic artery (SpA) (Fig. 1f). The SpA was divided at the distal dividing line of the pancreas, far enough from the origin of the SpA that dissection around the SpA and exposure of the DPA were prevented. The pancreas was divided together with the splenic vein using the Signia™ stapling system. Epithelial cells in the pancreatic tail stump showed no atypia on histopathology. Finally, 4.6 cm of the pancreatic body was preserved (Fig. 2a) and 10 mg of ICG was intravenously administered. The presence of fluorescence in the pancreatic remnant was definitively confirmed with a fluorescence camera (Fig. 2b). The reconstruction was done via a modified Child method with modified Blumgart pancreaticojejunostomy.

**Fig. 3:**
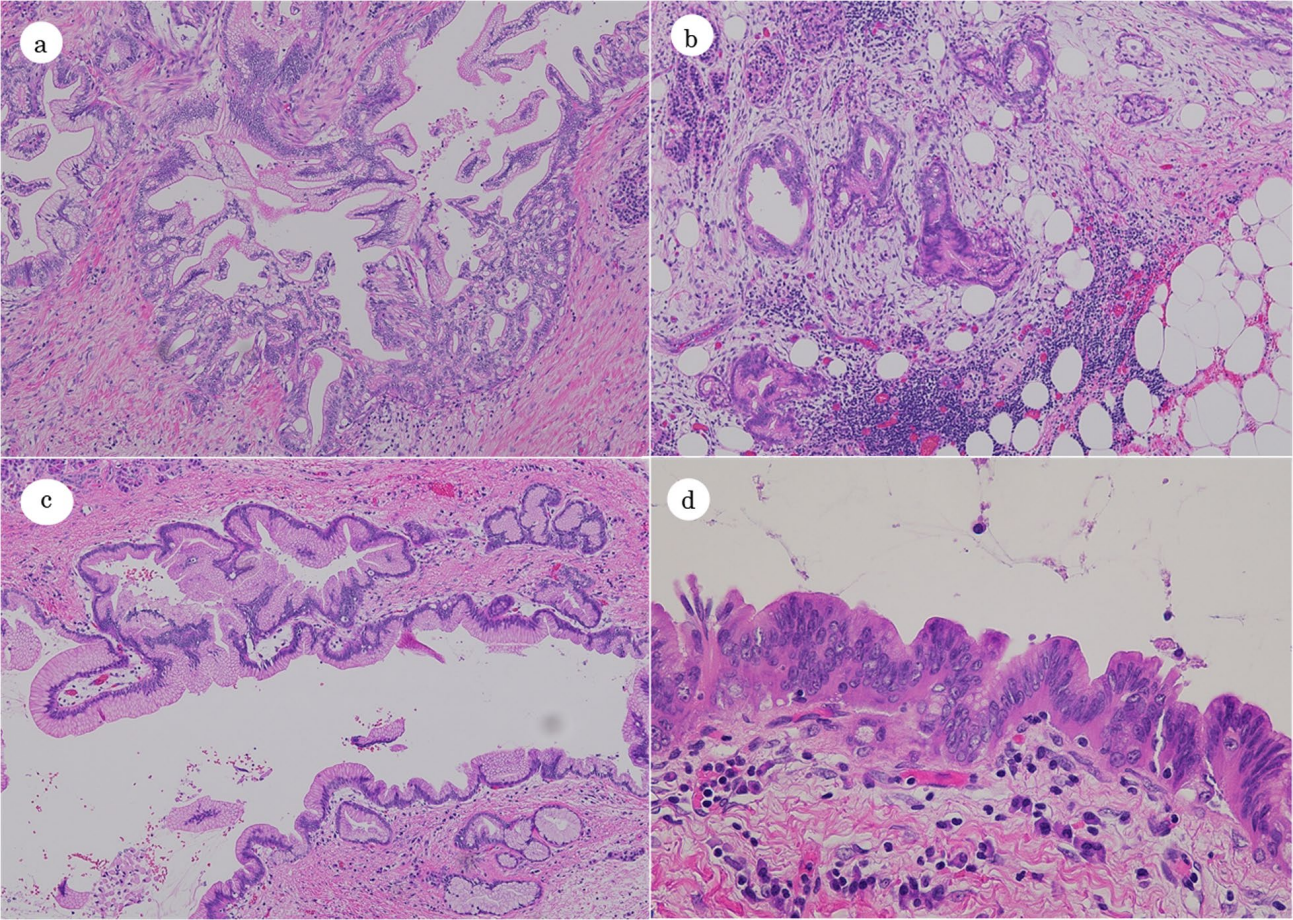
Pathological findings. The tumor in the uncus of the pancreas was pancreatic cancer, invasive ductal carcinoma (well to moderately differentiated; hematoxylin–eosin, original magnification 40× ; **a** invading neighborhood surrounding adipose tissue (hematoxylin–eosin, original magnification 40× ; **b** The tumor in the tail of the pancreas was histologically diagnosed as IPMN (low grade; hematoxylin–eosin, original magnification 40× ; **c** 400× ; **d** *IPMN* intraductal papillary mucinous neoplasm

Histopathological examination revealed that the tumor in the uncus of the pancreas was PDAC (pT1cN1M0, pStage 2B, UICC 8th) and that complete resection was achieved (Fig. 3a and b). The other tumor in the tail of the pancreas was found to be an intraductal papillary mucinous adenoma with mild atypia (Fig. 3c and d).

**Fig. 4:**
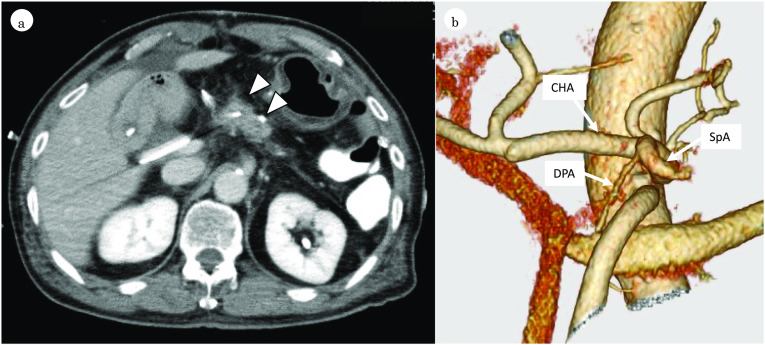
Postoperative CT revealed the enhancement of pancreatic remnant (arrowhead) (**a**). Postoperative 3D-CT showed that the DPA was successfully preserved (**b**). *DPA* dorsal pancreatic artery

The postoperative course was complicated by an International Study Group of Pancreatic Fistula (ISGPF) classification grade B pancreatic fistula from the distal stump, but the patient recovered well with conservative drain management. Postoperative CT examination showed that the pancreatic remnant was well preserved with good blood supply (Fig. 4a) and the DPA was preserved (Fig. 4b). The patient was transferred to a hospital 33 days after surgery. Serum C-peptide immunoreactivity (CPR) during fasting and 2 h after breakfast were 0.61 ng/ml and 0.27 ng/ml, respectively. Administration of an insulin preparation was necessary; however, blood glucose was relatively well-controlled and no symptomatic hypoglycemia occurred. At 2 months of follow-up, HbA1c level was 6.3%. No steatorrhea or malabsorption occurred when using pancreatic enzyme supplementation.

## Discussion

Multicentric tumors involving two or more pancreatic regions generally warrant TP, however, the metabolic consequences of TP are insulin-dependent DM and severe diarrhea as a result of the apancreatic condition [3]. Long-term, symptomatic hypoglycemia has been found to occur in 79–91% of patients who undergo TP [6, 7]. Miura et al. [4] first reported in 2007 the use of MSPP as an alternative to TP. Since then, several case reports have demonstrated that certain well-selected patients with multicentric tumors might benefit from MSPP as a safer, organ-sparing procedure [8–14]. The association between the remnant volume of the pancreas and postoperative exocrine and endocrine function has been evaluated [15, 16]. Ohzato et al. stated that even if the length of the pancreatic remnant is only 4 to 5 cm, good glucose tolerance may be maintained postoperatively [8]. In our case, the patient required an insulin preparation as an alternative to oral medications despite the pancreatic remnant being 4.6 cm. According to a literature review of 22 cases, postoperative insulin use was reported to be avoidable in 77% of patients who underwent MSPP [9], however, those patients had no history of DM. Meanwhile, nearly 80% of PDAC patients have either impaired glucose tolerance or evident DM [17]. The patient in the present case with PDAC also had a history of DM that was being treated with multiple medications, and postoperative additional insulin secretory function was extremely low, nevertheless, basal insulin secretory function was relatively maintained. Symptomatic hypoglycemia did not occur, glucose levels were well-controlled with insulin administration and HbA1c level was 6.3% at 2 months after surgery. It was unclear whether the objective pancreatic exocrine function was preserved or not; nevertheless, no steatorrhea or malabsorption occurred when using pancreatic enzyme supplementation.

To prevent postoperative pancreatic infarction after MSPP, the preservation of the feeding artery into the pancreas is necessary; particularly the DPA that feeds the middle segment of the pancreas [4, 10–12]. However, in most reports describing MSPP, it is unclear whether the DPA is preserved or not. The origin of the DPA is most frequently the proximal SpA, but DPA ramification is complex with many individual differences [18, 19]. The DPA was found to exist in all patients in an investigation of cadaveric specimens [20], however, the CT depiction rate of the DPA was reported to be 64–96.3% [18, 19]. Yamada et al. demonstrated that some small vessels that branch from the SpA feed the body of the pancreas, rather than the DPA. Therefore, several reports have proposed that lymph node dissection around the celiac axis or the SpA should be limited [10, 14]. In the current case, it was confirmed by the preoperative CT that the DPA originated from the proximal SpA and that the DPA could be preserved. Because the IPMN at the tail of the pancreas was a low-malignancy lesion, lymph node dissection around the SpA and the exposure of the proximal SpA and DPA were avoided for the preservation of the DPA and the small vessels from the SpA. It is important to avoid unnecessary dissection around the SpA and celiac axis and to understand the relationship between the origin of the DPA and the resection line of the distal pancreas in the preoperative CT.

In most case reports describing MSPP, postoperative CT revealed the maintenance of good blood supply to the pancreatic remnant, however, there are a few reports which also describe evaluating the perfusion to the pancreatic remnant intraoperatively. Clinical inspection of the pancreatic remnant or normal bleeding from the cut surfaces of the pancreas [13] are the simplest, least objective methods to confirm perfusion to the pancreatic remnant. Doppler ultrasonography can show the real-time arterial flow and evaluate the perfusion to the pancreatic remnant [8, 10]. However, its spatial resolution is inferior and its ability to show concealed arteries or information about venous perfusion or microperfusion is lacking [21]. As ICG administered intravenously remains within the intravascular space because it binds to plasma lipoprotein, ICG fluorescence represents a reliable tool for assessing microperfusion of the organ. The assessment of microperfusion using ICG fluorescence allows evaluation of the risks associated with anastomosis in colorectal, esophageal, and pancreatic surgeries [5, 22, 23]. With regard to pancreatic surgery, pancreas perfusion was investigated using ICG fluorescence for assessment of surgical risk after pancreaticoduodenectomy and was mostly detected within 30 s [23]. In the current study, the DPA was preserved by avoiding dissection around the proximal SpA and exposure of the DPA, therefore, perfusion to the pancreatic remnant could be confirmed immediately using intravenous injection of ICG dose of 0.2 mg/kg as previously reported [24]. For the first time to our knowledge, we describe the use of ICG fluorescence to confirm perfusion of the pancreatic remnant during MSPP.

## Conclusions

MSPP is a function-preserving procedure with satisfactory feasibility and effectiveness, however, the perioperative evaluation of perfusion to the pancreatic remnant is essential. ICG fluorescence may be useful to detect perfusion to the pancreatic remnant, especially when the DPA cannot be identified by preoperative CT or the origin of the DPA is not the SpA.

## Data Availability

The authors declare that all the data in this article are available within the article.

## Abbreviations

PDAC: Pancreatic ductal adenocarcinoma
IPMN: Intraductal papillary mucinous neoplasm
TP: Total pancreatectomy
DM: Diabetes mellitus
MSPP: Middle segment-preserving pancreatectomy
ICG: Indocyanine green
CA19-9: Carbohydrate antigen 19-9
HbA1c: Glycated hemoglobin A1c
CT: Computed tomography
EUS: Endoscopic ultrasonography
DPA: Dorsal pancreatic artery
SpA: Splenic artery
CPR: C-peptide immunoreactivity
